# Tumors recycle glucocorticoids to drive Treg-mediated immunosuppression

**DOI:** 10.1172/JCI173141

**Published:** 2023-09-15

**Authors:** Julian Swatler, Young-Jun Ju, Ana C. Anderson, Enrico Lugli

**Affiliations:** 1 Laboratory of Translational Immunology, IRCCS Humanitas Research Hospital, Rozzano, Milan.; 2 Gene Lay Institute of Immunology and Inflammation, Brigham and Women’s Hospital, Harvard Medical School, Boston, Massachusetts, USA.

## Abstract

Suppression of antitumor immunity is a prominent feature of the tumor microenvironment. In this issue of the *JCI*, Taves, Otsuka, and authors show that glucocorticoids (GCs), which are potent immunosuppressive hormones mainly produced by the adrenals, can be reconverted from their inactive form to active metabolites via the 11β-hydroxysteroid dehydrogenase type 1 (11β-HSD1) enzyme expressed by murine tumor cell lines. In the tumor microenvironment, GCs acted on CD4^+^ regulatory T cells to enhance their immunosuppressive function and promote tumor growth. The findings suggest that targeting GC recycling as a strategy for modulating tumor immunosuppression has the potential to improve therapeutic efficacy of immune checkpoint blockade.

## Glucocorticoids mediate immunosuppression

Immunosuppression is a prominent feature of cancer development, restraining antitumor effector T cell responses and limiting efficacy of therapeutic interventions. Immunosuppression can be mediated by multiple mechanisms, including immunosuppressive cells, such as CD4^+^ Tregs and suppressive myeloid cells, immunoregulatory cytokines, such as TGF-β, or enzymes, such as CD39 or arginase-1, that produce inhibitory metabolites. Recently, steroid hormones, namely androgens and glucocorticoids (GCs), have also been implicated in tumor immunosuppression (1), leading to CD8^+^ T cell dysfunction, prevention of effector differentiation (2), and diminished glycolytic metabolism (3), overall resulting in reduced antitumor activity. Moreover, GC receptor (GR) expression and signaling have been shown to upregulate PD-L1 and downregulate MHC class I expression by pancreatic ductal adenocarcinoma (PDAC) cells, enabling evasion of antitumor immunity (4). However, the potential origin of immunoregulatory GCs in the tumor milieu is not entirely clear.

The primary source of GCs in the body is the adrenals, where several enzymes sequentially break down cholesterol to produce active GCs — cortisol (in humans) and corticosterone (in mice). Recent reports have shown that this pathway can also be active in intratumoral leukocytes, such as monocytes/macrophages and T cells, leading to evasion of antitumor immunity (2, 5). Active GCs can be further inactivated into cortisone (or dehydrocorticosterone [DHC] in mice), but recycled back to active GCs by the enzyme 11β-hydroxysteroid dehydrogenase type 1 (11β-HSD1). Several organs express the 11β-HSD1 enzyme, potentially enabling alternative GC production (6, 7).

## GC modulation of Tregs in a tumor setting

In this issue of the *JCI*, Taves, Otsuka, and colleagues show that murine tumor cell lines express the enzyme 11β-HSD1 and could reconvert inactive GCs to their active metabolites, which in turn promoted tumor growth by potentiating the immunosuppressive capacity of CD4^+^ Tregs (6). Indeed, mouse tumor cell lines, such as melanoma B16, thymoma EL4, pancreatic adenocarcinoma Panc02, and colon carcinoma MC38, were able, with different efficiencies, to convert the inactive GC metabolite DHC to corticosterone in an 11β-HSD1–dependent manner in vitro.

**Figure 1:**
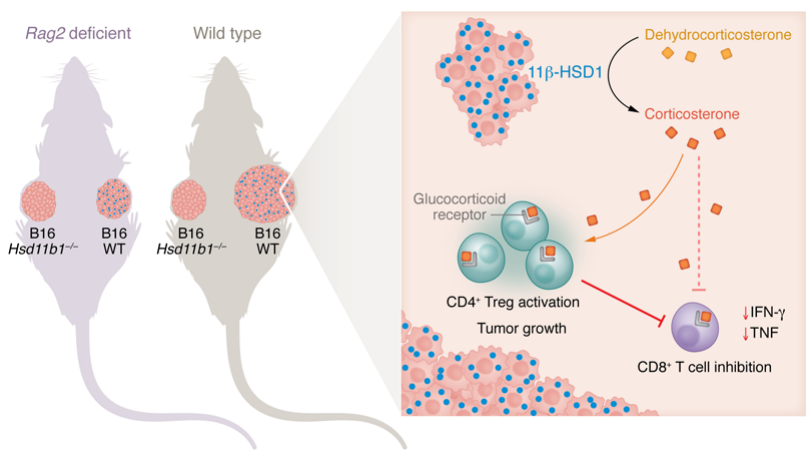
Tumors recycle GCs to drive Treg-mediated immunosuppression. The murine B16 tumor cell lines express 11β-HSD1 to convert inactive GCs to their active metabolites (6). Within the tumor microenvironment of immunocompetent mice, GCs act on CD4^+^ Tregs to enhance their immunosuppressive function (6) and on CD8^+^ T cells to impede function (2), thereby promoting tumor growth. 11β-HSD1–producing B16 tumor cells in *Rag2*-deficient mice, which lack T and B cells, cease to expand, suggesting that GCs act locally on adaptive immune cells to promote tumor growth.

To formally test the role of 11β-HSD1 (encoded by *Hsd11b1*) in tumor development, the authors used CRISPR/Cas9 to generate *Hsd11b1*-knockout cells and evaluated the growth of B16, Panc02, and MC38 tumors. *Hsd11b1* deficiency did not affect cell growth in vitro, but substantially delayed tumor growth in vivo in wild-type, immunocompetent mice independently from the expression of GR by tumor cells themselves (Figure 1). Moreover, expression of 11β-HSD1 led to a 3-fold increase in intratumoral levels of corticosterone. These data indicate that tumor-derived GCs did not act in an autocrine manner; rather they acted on the surrounding tumor immune microenvironment. Tumor-derived corticosterone was demonstrated to act locally, as wild-type and *Hsd11b1*-knockout B16 tumors that were injected in the contralateral flanks of the same animal exhibited differences in in vivo growth.

GCs predominantly influence T cell–mediated immunity in tumors (1, 2, 8). Indeed, when Taves, Otsuka, and authors implanted B16 into *Rag2*-deficient mice, which lack T and B cells, the influence of 11β-HSD1 on tumor growth was no longer visible, indicating that GCs act in a paracrine manner on lymphocytes to promote tumor growth (6). Analyses of the immune infiltrate in 11β-HSD1–deficient B16 tumors revealed expansion of effector memory CD8^+^ T cells, increased production of TNF and IFN-γ by CD8^+^ T cells, and finally, increased mTORC2/rictor levels in Tregs whose expression has been associated with attenuated suppressive function.

It has been widely recognized that Tregs are highly abundant in tumors and are markedly affected by GCs. In models of autoimmune (experimental autoimmune encephalomyelitis [EAE]) and allergic (airway hyperreactivity) inflammation, Tregs have been shown to be necessary for the antiinflammatory function of GCs, since dexamethasone, a synthetic GC, is unable to control inflammation in mice with GR-deficient Tregs (9). Along the same line, GCs drive Treg differentiation in the thymus and periphery, promote secretion of the immunosuppressive cytokine IL-10, and enhance Treg-mediated control of experimental colitis in mice (8). However, little is known about GC modulation of Tregs in a tumor setting.

To address this aspect, Taves, Otsuka, and authors used mice deficient in the GR specifically in the Treg lineage (*Nr3c1^Foxp3-cre^* mice) and found that B16 and Panc02 tumor growth was reduced. Moreover, when 11β-HSD1–deficient B16 was used, the effect of Treg-specific GR deletion was diminished, supporting the premise that GC recycling by tumor cells acts via Tregs to promote tumor growth. Curiously, the quantity of Tregs was unaffected in *Nr3c1^Foxp3-cre^* mice, implying the differences in tumor growth could be attributed to Treg functionality as opposed to homeostasis. Bulk RNA-Seq and gene set enrichment analysis revealed that tumor Tregs in *Nr3c1^Foxp3-cre^* mice adopted features of activated conventional T cells, whereas tumor Tregs from *Nr3c1^fl^* control mice resembled more activated Tregs. Although the suppressive capacity of Tregs was not assessed directly, these observations raise the possibility that in the absence of GC signaling, Tregs may become fragile and less suppressive (6, 10).

GC biogenesis pathways and their immunoregulatory functions are conserved between mice and humans. With this in mind, Taves, Otsuka, and colleagues mined The Cancer Genome Atlas (TCGA) and Oncomine databases of human tissues using bioinformatic approaches and found that, similarly to what occurs in mice, the *HSD11B1* gene is expressed across almost all tissues of the body. Crucially, *HSD11B1* expression was higher in both solid tumors and lymphomas compared with matched healthy adjacent tissue. Further, *HSD11B1* expression positively correlated with expression of effector Treg genes (*CCR8*, *CTLA4*, *ICOS*, *IL1R2)* and of genes encoding checkpoint receptors (*PDCD1*, *LAG3*, *HAVCR2*, *TIGIT*) (6). The latter is consistent with the direct transactivation of these genes by the GR (2). Accordingly, calculation of estimated frequencies of immune cell populations in TCGA data sets revealed a positive correlation of *HSD11B1* gene expression with Treg infiltration into tumors. However, in Taves, Otsuka, et al., Treg-specific deletion of GR in mice did not affect those Treg genes correlating with *HSD11B1* in humans which were generally expressed by highly immunosuppressive Tregs (11); overall Treg numbers were also unaffected (6). Hence, it remains possible that the correlation found between levels of *HSD11B1* and Treg infiltration in human cancers is an epiphenomenon reflecting the level of inflammation in the tissue rather than a direct causal relationship of GC signaling with Treg suppression and infiltration. Clarifying these aspects would have important implications for translation in humans.

## Implications and conclusions

GCs are potently immunosuppressive. Dampening their effect could have major consequences in reinvigorating the antitumor immune response; however, GCs are important for normal body homeostasis, making global targeting problematic (12). In this regard, the role of GC recycling by 11β-HSD1 in Treg-mediated immunosuppression indicates a possible avenue for therapeutic targeting. To complement the experiments in Taves, Otsuka, et al. in which 11β-HSD1 was deleted using genetic tools, the authors utilized two pharmacological inhibitors of 11β-HSD1 and showed attenuation of B16 melanoma tumor growth similar to that obtained by genetic deletion. Furthermore, pharmacological inhibition was tested in tumors developed from cell lines obtained from a genetically engineered melanoma model that reflects human triple wild-type (*BRAF/RAF/NF1*-WT) melanoma and its response to immunotherapy. Also, in this model, inhibition of 11β-HSD1 delayed tumor growth and, moreover, sensitized tumor cells to anti–PD-1 treatment, suggesting that GC inhibition may increase the effectiveness of immune checkpoint blockade in the clinic. These results align with the demonstration that loss of GC signaling in CD8^+^ T cells improves responses to anti-PD1 in mice bearing MC38-Ova^dim^ tumors (2). Thus, targeting of GC recycling rather than its biogenesis per se may provide an attractive avenue for improving therapeutic efficacy of immune checkpoint blockade, although systemic consequences and overall toxicity of this approach were not investigated in detail (6).

In summary, Taves, Osuka, et al. identified a mechanism of immunosuppression in the tumor microenvironment, namely, localized GC production by tumor cells via recycling of inactive metabolites that in turn act on tumor-infiltrating Tregs (6). These findings add to previously published results on the role of GC signaling in promoting CD8^+^ T cell dysfunction (2). They also pave the way for further studies aiming to interfere with GC metabolism in combination with immune checkpoint blockade to promote potent and durable T cell responses.

## Version 1 09/15/2023

Electronic publication
